# Antagonism between abscisic acid and gibberellin regulates starch synthesis and corm development in *Gladiolus hybridus*

**DOI:** 10.1038/s41438-021-00589-w

**Published:** 2021-07-01

**Authors:** Jingru Li, Shanshan Seng, Donglei Li, Fengqin Zhang, Yixuan Liu, Ting Yao, Jiahui Liang, Mingfang Yi, Jian Wu

**Affiliations:** Beijing Key Laboratory of Development and Quality Control of Ornamental Crops, Department of Ornamental Horticulture and Landscape Architecture, China Agricultural University, Beijing, China

**Keywords:** Plant physiology, Plant development

## Abstract

Understanding corm development in flower bulbs is of importance for securing the quality of cut flowers and propagation of commercial stocks. Gladiolus is one of the most popular bulb plants worldwide. Its corm development is characterized by starch accumulation. Previous research has shown that phytohormones (especially gibberellin (GA)) are involved in tuber development. However, the relationship between abscisic acid (ABA)/GA and starch during corm development remains unclear. To gain deeper insights into the biological process of corm development, we performed a detailed anatomical characterization of different stages of corm development and analyzed phytohormone levels. Our study showed that corm development is linked to hormones (ABA and GA) and carbohydrates (sucrose and starch). Exogenous hormone treatment and silencing of endogenous hormone biosynthesis genes indicated that ABA positively regulates corm development, while GA acts as an antagonist of ABA function. A sucrose synthase gene (*GhSUS2*) was shown to be involved in the antagonism between ABA and GA. *GhSUS2* was upregulated by ABA and downregulated by GA. The increase in the transcript level of *GhSUS2* coincided with the development of corm/cormels. Silencing of *GhSUS2* repressed corm development and starch accumulation. In conclusion, we propose that GhSUS2, an essential enzyme in sucrose degradation, is differentially regulated by ABA and GA and controls corm development in Gladiolus.

## Introduction

A storage organ is a portion of a plant specifically modified as a reserve of energy or water. This is an evolutionary strategy that maintains plant survival and propagation from generation to generation. The storage organs of geophytes are often found underground and are modified from the root, leaf, stem, or hypocotyl. They include bulbs, tubers, corms, rhizomes, and root tubers. Storage organs serve as sources of food (e.g., potato), commercial goods (e.g., *Fritillaria* spp.), and decorations for gardens (e.g., *Gladiolus* spp.). To date, the development of storage organs is poorly understood, except for potato. For wild potato species, tuberization is regulated by environmental factors, such as ambient temperature, photoperiod, and nitrogen^1,2^. Tuberization is stimulated under short-day conditions and promoted by low night temperatures^1^. Under long-day or high-temperature conditions, inhibition of tuberization is mediated by the CONSTANTS/FLOWERING LOCUS T (CO/FT) protein^3,4^. In hydroponically cultivated potatoes, tuberization is induced by withdrawing nitrogen from the nutrient solution, indicating that nitrogen has an inhibitory effect on tuberization^5^. In the context of phytohormones, GA dominates tuberization^6^. The GA level decreases when the stolon tip starts to swell, and excess GA inhibits the transition from stolon to tuber^7,8^. GA metabolism genes (*StGA20ox1* and *StGA3ox2*) have been shown to be involved in tuberization^9,10^. Cytokinins and jasmonic acid (JA) positively induce tuberization^11,12^. Aside from insights into the regulation of tuberization in potato, the physiological and molecular mechanisms of storage organ development in other species are poorly studied. Recently, by transcriptome and physiological analyses, a GA inhibitor was shown to stimulate the shoot-to-bulblet transition of lily in vivo, and starch was found to be a fundamental compound in this process^13^. However, direct genetic evidence of the role of hormones in bulb development remains elusive.

As the principal carbohydrate in storage organs, starch plays crucial roles in the development of storage organs, such as tubers and seeds^14–16^. Starch is an insoluble glucan comprised of amylopectin, amylose, and two polymers of glucose^17^. Sucrose is loaded from photosynthetic organs (e.g., leaves) and unloaded in nonphotosynthetic organs (i.e., sink organs) where sucrose is converted to starch for long-term storage in amyloplasts. Upon arriving in sink organs, sucrose is transported into sink cells via transporters or plasmodesmata^18,19^. In the cytosol, sucrose can be hydrolyzed by cytosolic invertase (CIN) to fructose and glucose or cleaved by sucrose synthase (SUS) to fructose and uridine diphosphate glucose (UDP-G). In the latter pathway, UDP-G can be further transformed to adenosine diphosphate glucose (ADP-G) by UGPase and AGPase^20^. ADP-G is the main substrate for starch biosynthesis in angiosperms. The glucosyl moiety is added to existing glucan chains by starch synthases^17^. Starch synthesis is regulated by phytohormones. In maize and potato, GA inhibits starch accumulation, while its inhibitor (chlorocholine chloride) significantly increases starch content^21,22^. In rice internodes, overexpression of ethylene receptors (ETHYLENE RESPONSE2/ETR3) induces the accumulation of starch granules^23^.

SUS is a glycosyltransferase that catalyzes the reversible transfer of a glucosyl moiety between fructose and UDP-G^24^. UDP-G is used to biosynthesize starch or cellulose in plants^25^. Selective phosphorylation of sucrose synthase isoforms results in enhanced sucrose degradation^26^. SUS promotes vegetative growth, early flowering, plant biomass accumulation, and the response to low oxygen^27–29^. Moreover, SUS decomposes sucrose in sink organs, generating a sucrose gradient flow from source to sink, i.e., the flow provides pressure for the transport of sucrose from the phloem to the sink and ensures the continuous supply of sucrose to the sink in potato. Reducing SUS activity in potato results in reduced tuber dry weight and a lower content of starch^30^.

Although much is known about sink organs in model plants, it has become clear that research on model plants alone will not provide adequate information for the improvement of starch accumulation in all plant species. Indeed, factors controlling starch metabolism differ among species and organs^17^. Therefore, it is worthwhile to study the role of starch in the development of storage organs in nonmodel plants. Gladiolus is one of the most widely planted geophytes worldwide, and its corm is the only plant stock and propagation tissue used for commercial cultivation. Corm degradation, which leads to decreased cut flower production and propagation yield, is one of the most severe problems in Gladiolus cultivation. The gladiolus corm is a specialized underground organ consisting of an enlarged stem axis with distinct nodes and internodes and enclosed by dry, scale-like leaves (tunics). A new corm is generated each growing season over the mother corm. Meanwhile, cormels are produced at the tips of branched stolons that develop from buds located at the base of the new corm^31^. Corm development is tightly related to carbohydrates. During the rapid expansion of corm and cormels, starch and sugar levels increased sharply^32^. Recently, we have shown that induction of starch metabolic genes (*the small/large subunit of ADP-glucose pyrophosphorylase*; *GhAGPS1*/*GhAGPL1*) stimulates corm development and cormel numbers^33,34^. Additionally, JA could promote corm expansion, and the expression pattern of *GhLOX1* (*LIPOXYGENASE*), a key JA biosynthesis gene, coincides with corm development^35^. Whether other hormones participate in corm development and how hormones regulate carbohydrates in corms are still not clear.

Here, we show that the levels of starch and endogenous ABA increase sharply during the transition from stolon to cormel, while the GA_3_ level decreases. Interestingly, exogenous ABA promotes corm weight and cormel numbers, and GA_3_ represses the stolon-to-cormel transition. Furthermore, decreasing the ABA level in the corm by silencing *GhNCED* dramatically inhibited starch accumulation and the expansion of corms, which was the opposite of the phenotype observed in *GhGA20ox*-silenced corms. We show that *GhSUS2* expression is induced by ABA but inhibited by GA_3_. *GhSUS2* is expressed mainly in sink organs (corms and cormels) and upregulated during corm development. Silencing *GhSUS2* in corms impairs corm development. Hence, we propose that ABA and GA play antagonistic roles in corm development by differentially regulating *GhSUS2* expression and starch synthesis in Gladiolus.

## Results

### Endogenous ABA and GA levels, along with starch accumulation, change dramatically during Gladiolus cormel development

**Fig. 1:**
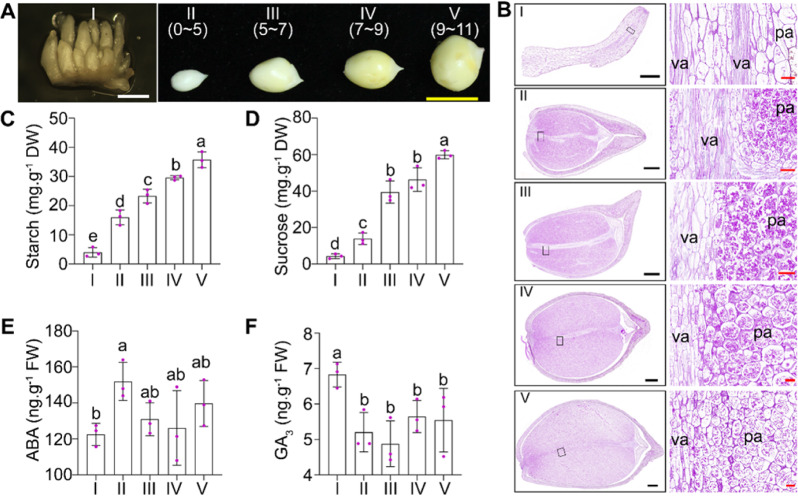
Carbohydrate and endogenous hormone levels change dramatically during Gladiolus cormel development. **A** Early cormel development is classified into 5 stages. I: stolons before swelling of the tips; II: pale cormels (*Φ* = 0–5 mm); III: cormels that have turned yellow (*Φ* = 5–7 mm); IV: enlarged cormels (*Φ* = 7–9 mm); cormels, as planting stocks (*Φ* = 9–11 mm). White bars = 1 mm, and yellow bars = 1 cm; **B** Sections of stolons and cormels at different stages stained by PAS. Va: vascular cells; pa: parenchyma cells. Black bars = 100 μm, red bars = 50 μm. Starch (**C**) and sucrose (**D**) levels at the different cormel development stages (*I*–*V*). Endogenous ABA (**E**) and GA_3_ (**F**) levels at the different cormel development stages (*I*–*V*). Averages of three biological replicates ± SDs (cormels/stolons from three different plants per biological replicate) are shown. Different letters represent statistically significant differences at *p* < 0.05 (one-way ANOVA and Tukey HSD post hoc test)

Early cormel development in Gladiolus can be divided into 5 stages (Fig. 1A): stolons, with a tip that is not swollen (I); pale cormels (II; 0–5 mm in diameter); yellow cormels (III; 5–7 mm in diameter); expanded cormels (IV; 7–9 mm in diameter) and cormels that become planting stocks in commercial cultivation (V; approximately 1 cm in diameter). To characterize the different stages of cormel development (I–V), we observed cells and sugar distribution by periodic acid-Schiff (PAS) staining. The results showed that parenchyma cells and vascular cells in stolons (stage I; Fig. 1B) accumulated or transported fewer sugars than formed cormels (stages II–V). After cormels formed at the tips of stolons, parenchyma cells started to accumulate sugars that were delivered from the mother corm (Fig. 1B). The distribution of sugars in cormels was not equal, with the bottom of cormels accumulating more sugars (Fig. 1B). At later stages (IV to V), the parenchyma cells were much larger, and the vascular cells accumulated sugars at the tip (Fig. 1B).

Next, we analyzed the starch and sucrose levels at the different developmental stages. The levels of both starch and sucrose were low in stolons but increased gradually in cormels from stage II to V (Fig. 1C, D).

As phytohormones regulate plant development, including germination, vegetative development, and reproductive development^36^, we quantified phytohormone levels during cormel development. When corms formed at the tips of cormels (transition from I to II), the endogenous ABA level increased sharply, while the GA_3_ level decreased (Fig. 1E-F). When cormels developed from III to V, endogenous ABA was maintained at a relatively high level (although not significantly higher than the level at stage I), while GA_3_ remained at a relatively stable lower level than the level at stage I (Fig. 1E, F). These results indicate a dramatic increase in sucrose and starch levels during corm development, accompanied by a transient increase in the ABA level and a decrease in the GA level.

### Exogenous ABA and GA affect the development of Gladiolus corm and cormels

**Fig. 2:**
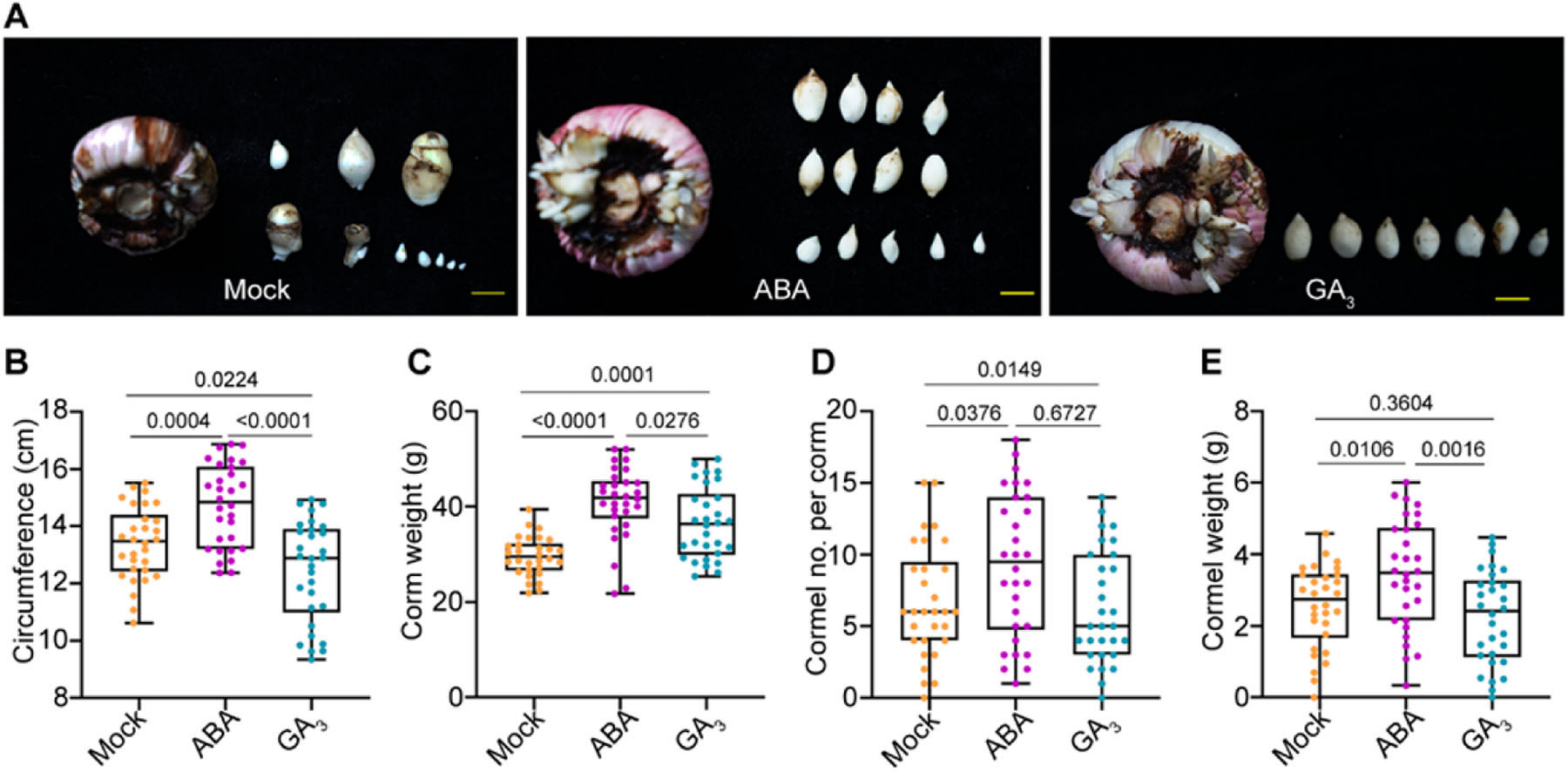
Gladiolus corm and cormel development was affected by ABA and GA_3_. **A** Phenotypes of corm and cormels after 6 weeks of treatment with ABA (0.5 mg/L) and GA_3_ (0.5 mg/L). Yellow scale bars = 1 cm. The circumference (**B**) and weight (**C**) of mother corms under different treatments**. D** Cormel numbers per plant under different treatments. **E** Total cormel yield per plant under different treatments. The data were collected after 6 weeks of treatment. The experiments were performed with three biological replicates (10 corms per biological replicate). The significant differences between the mock and treatments were determined by Tukey’s multiple comparison test. The *P* value is indicated above the black line

Given the opposite changes in ABA and GA_3_ levels during the transition from stage I to II (Fig. 1E, F), we hypothesized that these hormones play opposite roles in Gladiolus cormel formation and enlargement. To test our hypothesis, we treated plants with ABA and GA_3_ at 10 weeks after planting (WAP). Usually, cormels are largely formed at 14 WAP. After 6 weeks of treatment, plants under ABA treatment had larger mother corms (Fig. 2A–C). Moreover, ABA also significantly promoted the formation and development of cormels, resulting in more cormels and higher yield (Fig. 2D, E). Plants subjected to GA_3_ treatment had a slightly smaller circumference, but their corms were much heavier (Fig. 2B-C). GA_3_ played a negative role in corm formation by reducing the number of cormels (Fig. 2D). Taken together, the results show that ABA plays a positive role in the formation and development of cormels and corm development, while GA negatively affects the formation of cormels.

### Silencing ABA and GA biosynthesis genes affects Gladiolus cormel development

**Fig. 3:**
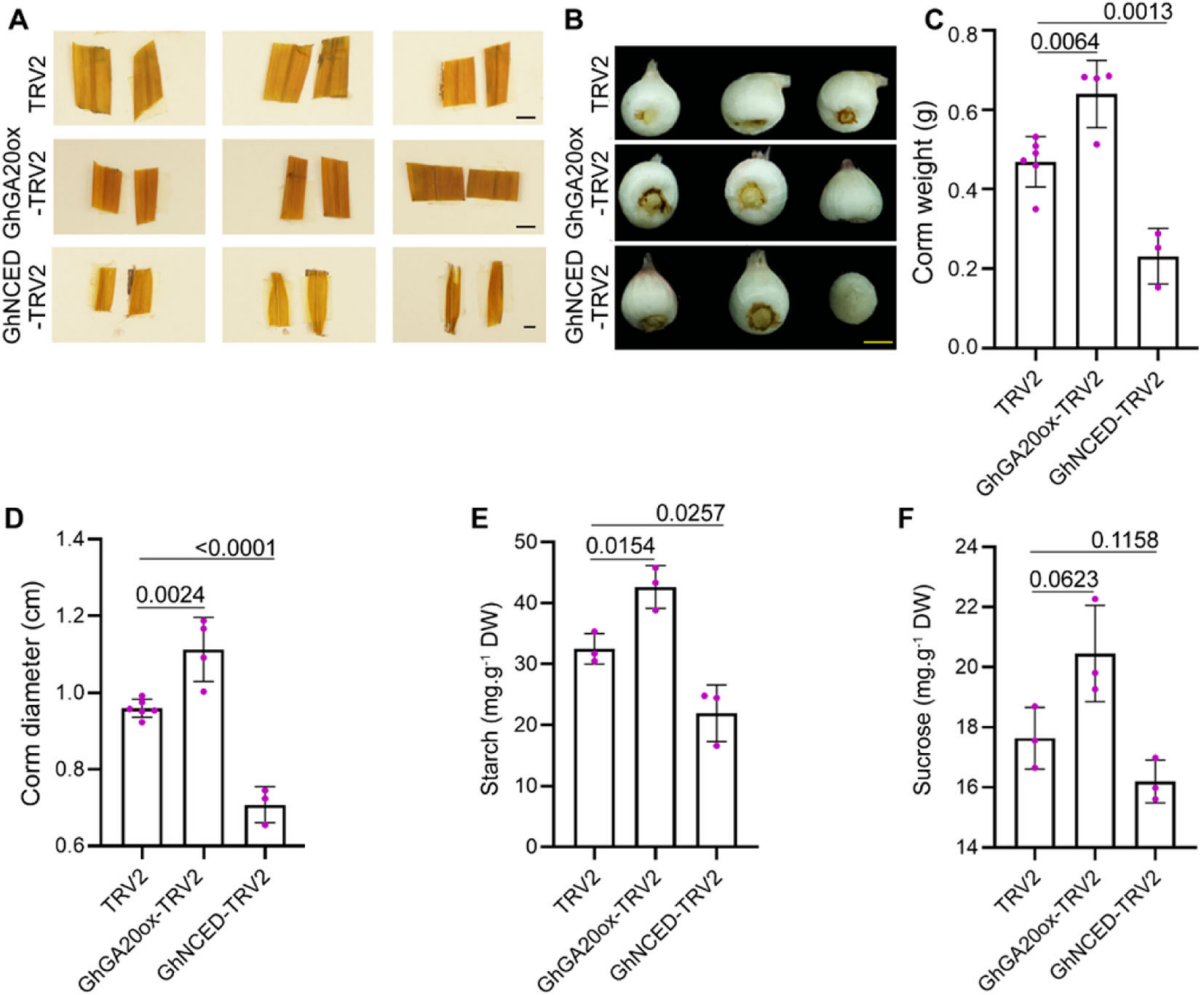
*GhNCED* and *GhGA20ox* regulate corm development and starch biosynthesis. **A**. Iodine staining of 2-month-old gene-silenced leaves. A darker color indicates a higher level of starch. Bars = 1 cm. **B**. Silencing of *GhNCED* or *GhGA20ox* affected corm development. The image was taken 4 months after planting. Bars = 0.5 cm. Measurements of corm weight (**C**) and diameter (**D**) in 4-month-old silenced plants. **E**. Silencing of *GhNCED* or *GhGA20ox* affected starch content in 4-month-old corms. **F**. Sucrose content at 4 months of age in gene-silenced corms. Error bars represent the SD of different silenced lines. Significant differences were determined by Tukey’s multiple comparison test. The P value is indicated above the black line

To further address the role of ABA and GA in cormel development, we silenced ABA and GA biosynthesis genes in cormels. Silencing GhNCED (NINE-CIS-EPOXYCAROTENOID DIOXYGENASE), a key enzyme in ABA biosynthesis in Gladiolus cormels, led to reduced ABA content (Fig. S1), increased GA_3_ content (Fig. S1) and early sprouting^37^. Here, we found that silencing *GhNCED* in corms before planting also reduced the starch content in leaves (Fig. 3A) and led to the generation of smaller and lighter corms (Fig. 3B–D). Conversely, silencing the GA biosynthesis gene *GhGA20ox* decreased GA_3_ levels and had the opposite effect, promoting the development of enlarged and heavier corms (Fig. 3B–D; Fig. S1). Furthermore, the starch content in *GhNCED*-silenced corms was dramatically lower than that in the control, while it was much higher in *GhGA20ox* (*gibberellin 20-oxidase*)-silenced corms (Fig. 3A, E, F). The sucrose levels did not change as obviously as those of starch in the silenced corms. These results, taken together with the previous results (Figs. 1 and 2), led us to conclude that ABA has positive effects, while GA has negative effects, on corm development. The effect of ABA and GA on corm development may be correlated with starch.

### Expression of *GhSUS2* is correlated with Gladiolus corm development and regulated by ABA and GA

Previous work has shown that SUSy, not INV, is the dominant active enzyme in actively growing sink organs in geophytes, such as potato tuber and cassava roots^19,38,39^. Moreover, SUSy is essential for sink strength, especially in starch-accumulating organs^19^. To identify corm-expressed *GhSUSs*, we screened our cormel transcriptome database^40^. We found six unigenes expressed in cormels, of which GlaUn069031 was the most abundant (Fig. S2A). GlaUn069031 has a sequence similar to those of the homologous genes in Arabidopsis (*AtSUS2*) and rice (*OsSUS2*), so we named it *GhSUS2* (Fig. S3A). GhSUS2 shared a conserved Ser-phosphorylation site at the N-terminus of the amino acid sequence (Fig. S3B).

**Fig. 4:**
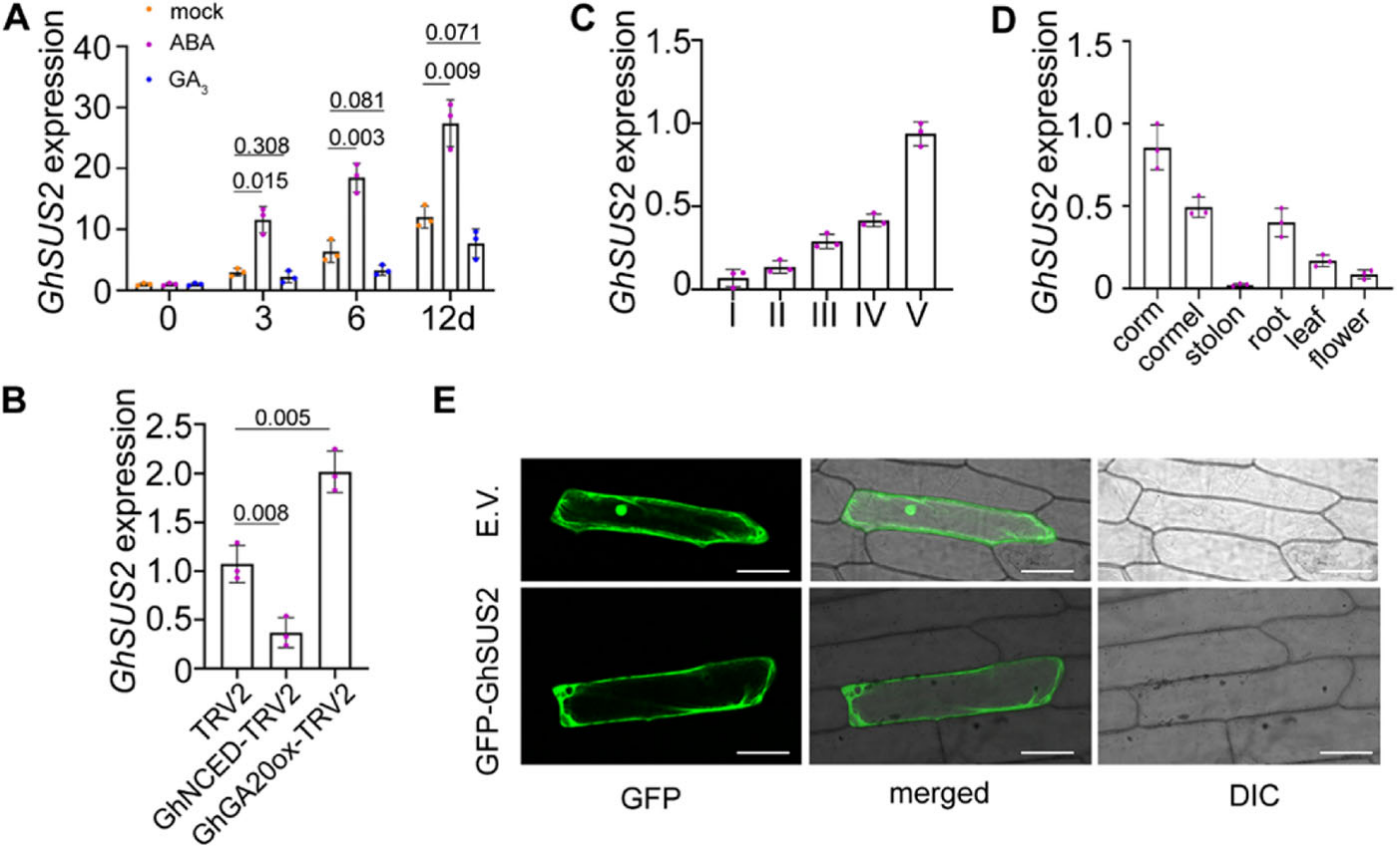
Expression of *GhSUS2* increases during Gladiolus corm development and is differentially regulated by ABA and GA. **A** qRT-PCR showing increased *GhSUS2* transcript levels under ABA (0.5 mg/L) treatment and slightly decreased transcript levels under GA_3_ (0.5 mg/L) treatment. **B** Differential expression of *GhSUS2* in GhNCED-TRV2 and GhGA20ox-TRV2 corms. **C** Increased *GhSUS2* transcript levels during corm development. **D** Expression pattern of *GhSUS2* in different organs. Averages of three biological replicates ± SDs (*n* = 3) are shown. Significant differences were determined by Tukey’s multiple comparison test. The *P* value is indicated above the black line. **E** Subcellular localization of GFP-GhSUS2 in onion epidermal cells. pSuper: GFP (E.V.; empty vector) was used as the control. Bar = 100 μm

Given that ABA and GA affected starch synthesis in Gladiolus (Fig. 3), we investigated the relationship between these two hormones and *GhSUS2*. First, we explored the expression pattern of *GhSUS2* in corms treated with ABA and GA_3_. Quantitative RT-PCR results revealed that *GhSUS2* expression was induced by ABA and slightly repressed by GA_3_, although the change was not statistically significant (Fig. 4A). We then tested the transcript level of *GhSUS2* in *GhNCED*- and *GhGA20ox*-silenced corms. The results showed that *GhSUS2* expression was decreased in *GhNCED*-silenced corms but increased in *GhGA20ox*-silenced corms (Fig. 4B). Altogether, our data indicate that *GhSUS2* expression is induced by ABA and repressed by GA.

To test the role of GhSUS2 in cormel development, we analyzed its expression pattern in different stages of cormel development (*I*–*V*) and in different organs. *GhSUS2* was highly expressed in sink organs (corms and cormels), and its expression level gradually increased with cormel development (Fig. 4C, D). In addition, we also tested *GhSUS2* expression in cormels at different growth points. The cormel was formed starting from 10 weeks after planting (WAP), developed until 26 WAP, and dried at room temperature for 4 additional weeks. The expression level of *GhSUS2* closely matched the level of sucrose accumulation in cormels (Fig. S2B, C).

Plant sucrose synthase isozymes are mainly located in the cytosol or adjacent plasma membrane^19^. To trace the protein localization, GFP was fused in frame with GhSUS2 (pSuper: GFP-GhSUS2). A green fluorescence signal of GFP-GhSUS2 was observed in the cytosol (Fig. 4E), showing similar subcellular localization as its homologs in potato^25^. This finding suggests that GhSUS2 is a cytosolic SUS.

### Silencing of *GhSUS2* represses Gladiolus corm development by decreasing starch content

**Fig. 5:**
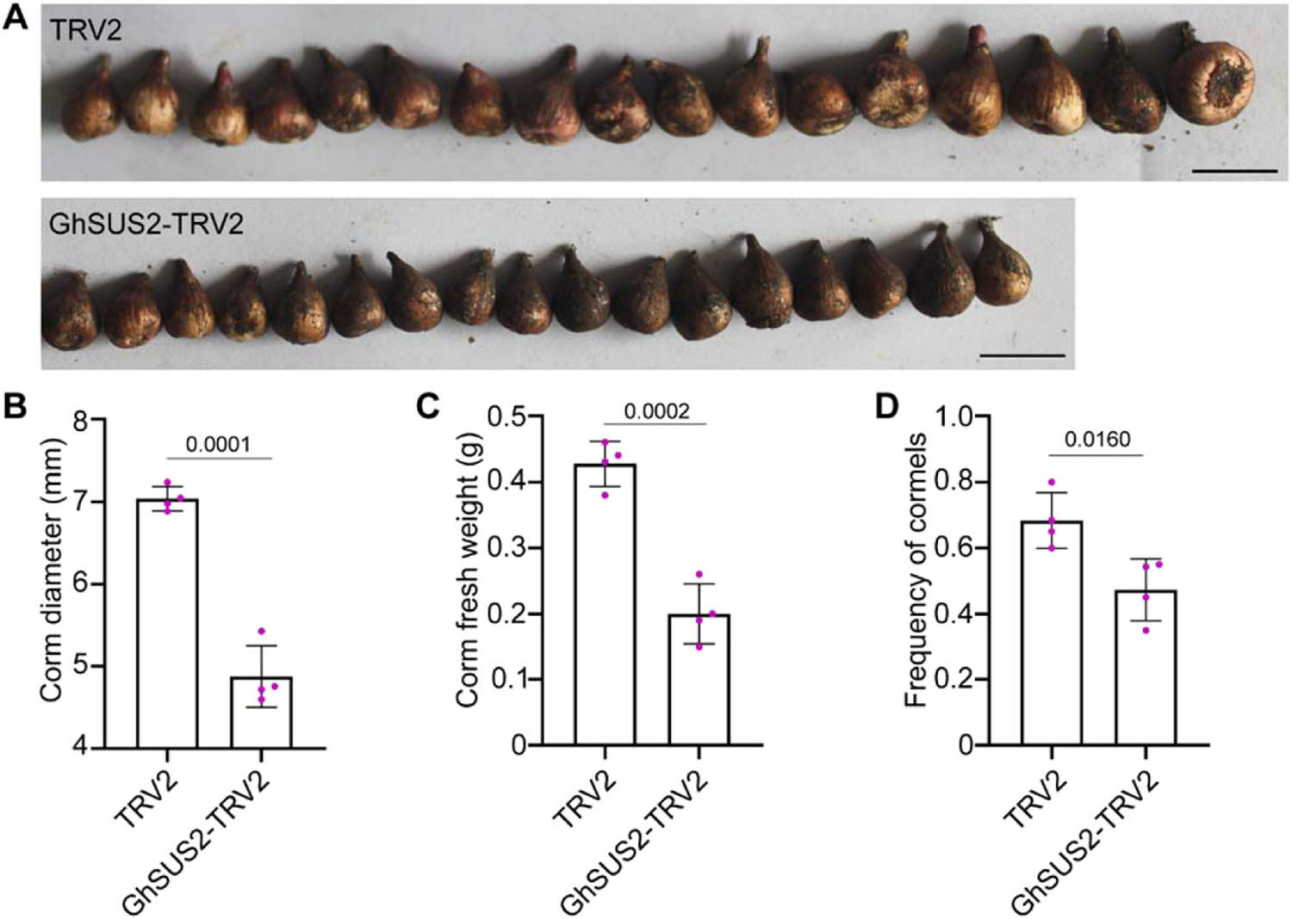
Silencing of *GhSUS2* represses Gladiolus corm development. **A** Silencing of *GhSUS2* in Gladiolus resulted in smaller corms than those observed in the control. The phenotype was observed four months after planting, and 17 representative lines are shown. Black scale bar = 1 cm. Silencing of *GhSUS2* in Gladiolus reduced the corm diameter (**B**) and corm fresh weight (**C**). **D**. The cormel frequency in *GhSUS2*-silenced plants was lower than that in the control. **B** to **D** Averages of four biological repeats, ± SDs (*n* = 24). Significant differences were determined by the two-sided *t*-test. The *P* value is indicated above the black line

As the expression of *GhSUS2* is correlated with starch synthesis and corm development, we reasoned that corm development should be regulated by *GhSUS2*. To test our hypothesis, we silenced *GhSUS2* in corms before planting them. After four months of growth, the silenced plants were dug out, and the corm size, corm weight, and cormel frequency were measured. Notably, the corm diameter in *GhSUS2*-silenced plants was significantly smaller than that in the control (Fig. 5A, B). The fresh weight of silenced corms was also smaller than that of the control (Fig. 5C). In addition to its effects on corm development, *GhSUS2* regulated cormel formation. The cormel frequency in the silenced plants was lower than that in the control (Fig. 5D).

**Fig. 6:**
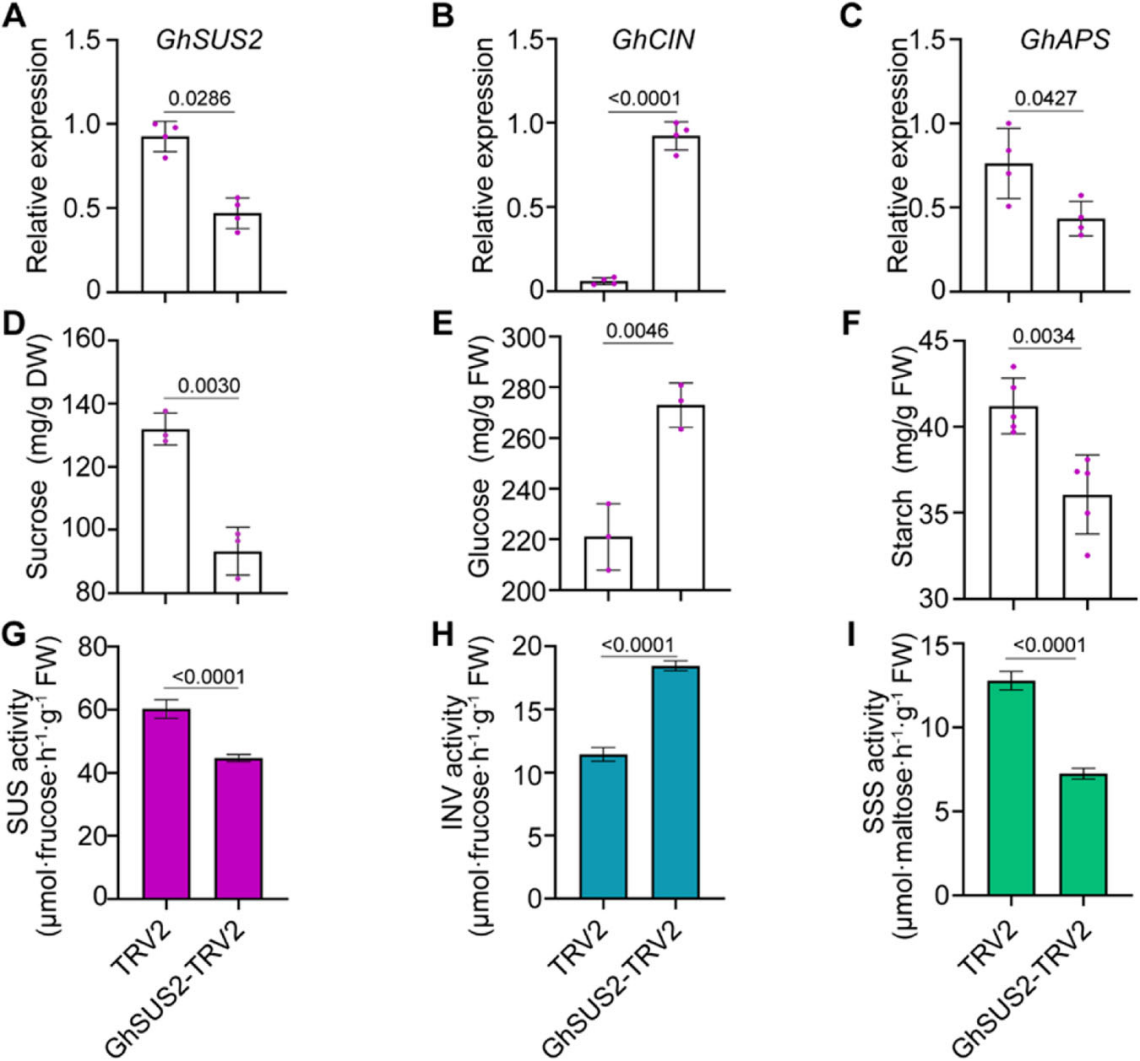
Silencing of *GhSUS*2 represses starch synthesis in corms. **A** Expression of *GhSUS2* in silenced corms. **B** qRT-PCR results showing an increase in the *GhCIN* transcript level in *GhSUS2*-silenced corms. **C** Downregulation of *GhAPS* expression in *GhSUS2*-silenced corms. **D** Decreased sucrose content in *GhSUS2*-silenced corms. **E** Accumulation of glucose in *GhSUS2*-silenced corms. **F** Repression of starch synthesis in *GhSUS2*-silenced corms. The enzyme activity of sucrose synthase (**G**) or soluble starch synthase (SSS; **I**) was decreased in *GhSUS2*-silenced corms. **H** The enzyme activity of invertase was increased in *GhSUS2*-silenced corms. Averages of three to five biological replicates ± SDs are shown. Significant differences were determined by the two-sided *t*-test. The *P* value is indicated above the black line

To better understand the role of GhSUS2 in corm development, we determined the expression pattern of key genes in starch biosynthesis in *GhSUS2*-silenced corms (Fig. 6A). Cytosolic invertase degrades imported sucrose to glucose and fructose in the cytosol^41^. Here, we found that the transcript level of *GhCIN* was sharply increased in the silenced corms, and accordingly, invertase activity was increased (Fig. 6B, H). The glucose content was higher in the GhSUS2-silenced corms, but the sucrose content was lower (Fig. 6D, E). In previous research, *GhAPS* has been shown to play a positive role in regulating corm development and starch synthesis^33^. qRT-PCR showed that *GhAPS* was downregulated when *GhSUS2* was silenced (Fig. 6C). Additionally, both sucrose synthase and soluble starch synthase had lower activity in *GhSUS2*-silenced corms than in the control (Fig. 6G, I). For these reasons, the starch content in *GhSUS2*-silenced corms was lower than that in the control (Fig. 6F). Overall, we concluded that GhSUS2 promotes corm development by modulating starch synthesis.

## Discussion

**Fig. 7:**
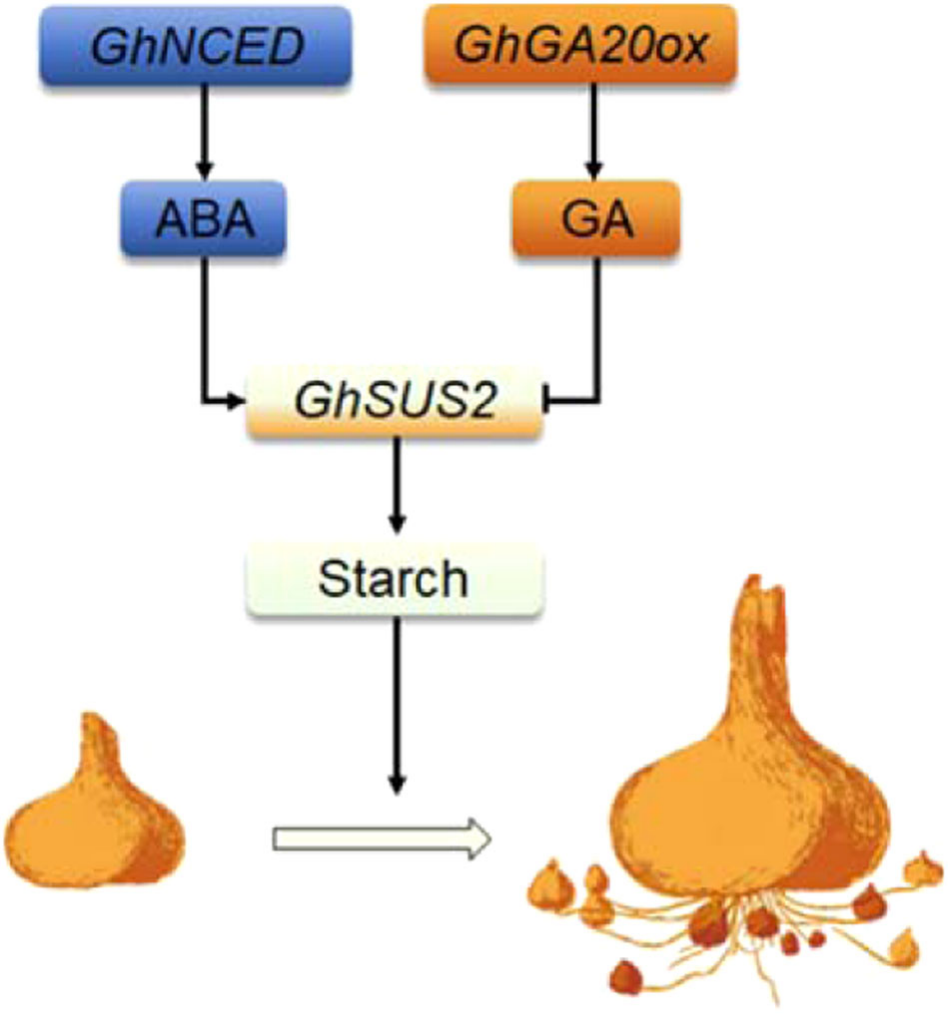
Model of Gladiolus corm development regulated by abscisic acid and gibberellin. ABA positively regulates corm development by enhancing *GhSUS2* expression and starch synthesis. Repressing *GhSUS2* decreases starch content and leads to the formation of small corms. GA has the opposite effect on *GhSUS2* expression and starch synthesis and causes a decrease in the number and size of cormels. Silencing *GhNCED* or *GhGA20ox* in corms had opposite effects on *GhSUS2* expression, starch accumulation and corm development

Starch serves as the main storage compound in higher plants and accumulates in sink organs (e.g., seeds, bulbs, roots, and buds) before the end of each life cycle. Transcriptome analysis in potato and Lilium suggests that sucrose and starch metabolism is involved in the tuberization and the shoot-to-bulblet transition^13,20,42–44^. In temperate zones, most plants enter dormancy during winter. ABA promotes seed dormancy, bud dormancy and bulb dormancy. Indeed, the ABA level gradually increases during the development of dormant organs (before they become dormant)^45–47^. However, the link between ABA and starch at this developmental stage is less well understood. Here, we found that ABA plays a positive role in corm development and cormel development. While ABA promotes starch synthesis by upregulating *GhSUS2* expression, GA has the opposite effect. Furthermore, we showed that starch synthesis positively regulates corm development. We propose that ABA promotes the development of corms in Gladiolus by increasing starch synthesis through upregulation of *GhSUS2*, while GA has the opposite effect (Fig. 7).

ABA and GA are two classic hormones in plants that antagonistically regulate several plant developmental processes, including seed maturation, seed dormancy and germination, primary root growth, and flowering time^48–50^. However, GA does not always function as an antagonistic hormone to ABA, e.g., GA inhibits corm dormancy release in the early stage of Gladiolus dormancy release, which is similar to the role of ABA^45,51^. Here, we provide evidence that ABA and GA antagonize corm development in Gladiolus: (i) during the transition from stolon to cormel, ABA production was induced and GA production was decreased (Fig. 1); (ii) opposite phenotypes of corm circumference and cormel numbers were observed following treatment with ABA or GA (Fig. 2); (iii) silencing *GhNCED* or *GhGA20ox* had opposite effects on corm development (Fig. 3). Notably, we found that GA_3_ could increase corm weight and the uniformity of cormels (Fig. 3). This might have been caused by cell expansion under GA treatment^52^. GA was shown to promote stolon elongation and reduce the activity of ADP-G^1,53^. Moreover, GA could also regulate tuber formation by mediating the FT/CO pathway^3^. However, JA and ABA do not seem to be effective hormones in tuberization^1^. In a recent study, ectopic expression of *AtABF4* (*ABRE BINDING FACTOR 4* in Arabidopsis) in potato caused GA-defective phenotypes and significantly increased the number and weight of the tubers obtained, suggesting that ABA/GA cross-talk may be involved in tuberization^6^. Although potato tuberization is regulated by the crosstalk between ABA and GA, the effects of ABA are always mediated by a change in the levels of GA, which is ultimately the master regulator of tuberization^6,54,55^. Previously, JA was reported to promote corm expansion in Gladiolus^35^, suggesting that the effects of hormones may be species dependent.

In this study, we showed that ABA increases the starch content in corms and promotes corm development and stolon-to-cormel transition. Typically, discourse on tuber development revolves around gibberellins. In the regulation of Gladiolus corm development and starch/sucrose levels, the effect of ABA was more obvious than that of GA, suggesting that ABA plays a more prominent role in corm development. *GhNCED* is most abundant in the stolon at the flowering/blooming stage when corms start to emerge^37^. This is in agreement with the fact that during the cormel transition, the levels of ABA and starch sharply increased (Fig. 1). NCED is a key enzyme in ABA biosynthesis and is widely involved in plant development and abiotic stress^56,57^. Unlike its role in model plants such as Arabidopsis, rice, and tomato, the role of NCED in modified organs is less well known. Here, we characterized the role of *GhNCED* in sink organs (corm and cormels) and starch synthesis (Fig. 3). Moreover, we found roles for ABA in promoting starch accumulation after stolon swelling, in cormel transition and in corm dormancy^37,45^.

### GhSUS2 positively regulates corm development

Sucrose synthase was discovered in beets, sweet sorghum, and pea seeds in 1955^58^. Most SUS members are found in the cytosol, and some are associated with the plasma membrane. A few SUS isoforms are found in the cell wall and other organelles (e.g., vacuole membrane, cytoskeleton, mitochondria, and Golgi apparatus)^19,59–62^. SUS is the primary active enzyme involved in the breakdown of sucrose in sink organs, enhancement of sink strength, and active growth of sink organs^19^. SUS is highly expressed in storage organs such as seeds, fruits, and taproots and is often positively correlated with starch content and fruit size^63–65^. In potato, reducing SUS by an antisense technique markedly decreased the starch content and tuber yield^63^. Here, we found that GhSUS2 is localized in the cytosol and that the expression of *GhSUS2* is positively related to the early developmental stage of cormels and starch accumulation (Fig. 1C, D, E; Fig. S2B). Silencing of *GhSUS2* in corms resulted in lower levels of *GhAPS*, sucrose and starch and reduced SSS enzyme activity (Fig. 6). Intriguingly, we found that INV activity and glucose content were increased in *GhSUS2*-silenced corms (Fig. 6). As SUS activity was reduced in the silenced corm, INV might have taken over sucrose degradation, resulting in increased glucose level. It has been shown that a high concentration of sucrose can induce tuberization in potato and bulb formation in onion^7,66^. In our *GhSUS2*-silenced corms, the sucrose level was also lower than that in the control (Fig. 6). Moreover, ectopic expression of *GhSUS2* in Arabidopsis could promote root elongation on sucrose-containing media (Fig. S4), suggesting that GhSUS2 could accelerate sucrose degradation in cells. In *GhSUS2*-silenced corms, there was a small decrease in starch content, suggesting that silencing *GhSUS2* may not only reduce starch biosynthesis but also slow starch degradation or conversion. Given this evidence, we speculate that GhSUS2 is involved in degrading sucrose in the cytosol and in converting sucrose to starch to promote the sucrose flow from source organs to sink organs.

In plants, the relationship between ABA and sucrose synthase is variable depending on the species. In pea, ABA induces a decline in nitrogen fixation in a manner that is independent of SUS^67^. In rice, ABA treatment shows a positive relationship between SUS and grain filling^68^. How does ABA relate to SUS in Gladiolus corm development? We utilized both exogenous ABA treatment and silencing of the ABA synthesis gene *GhNCED* to investigate the effects of ABA. Both assays showed that ABA increases the transcription of *GhSUS2* and starch in corms. Recently, a bZIP family member, AtABF4, was demonstrated to increase tuber yield through ABA-GA crosstalk regulation^49^. It will be interesting to investigate how ABA signaling-related transcription factors are involved in regulating GhSUS2 in Gladiolus in future work.

In conclusion, we showed that GhSUS2, an essential enzyme in the starch biosynthesis pathway that mediates the antagonism of ABA and GA, plays an important role in corm development in Gladiolus.

## Material and methods

### Plant materials and treatments

The Gladiolus cultivar ‘Rose Supreme’ was planted in the Science Research Garden at China Agricultural University. For tracking corm development, stolons and cormels (*Φ* = 0–5, 5–7, 7–9, 9–11 mm) were sampled at 10 WAP. For exogenous hormone treatment, uniform corms (6 cm in circumference and 3.0–3.8 g) from plants at 10 WAP were irrigated with ABA (0.5 mg/L; Solarbio, Beijing, China), GA_3_ (0.5 mg/L; Solarbio, Beijing, China), or water (the control) every 3 days. The plants were lifted after six weeks of treatment. Three biological replicates were examined (10 corms per biological replicate). For counting cormels, the expanded stolons, which were empty inside and not real cormels, were not included.

### Measurement of starch, glucose, and sucrose

Gladiolus corms or stolons (50 mg) were ground in powder by liquid nitrogen. The extraction and quantification of starch, glucose, and sucrose were performed as described by Fan et al.^69^.

### Measurement of ABA and GA_3_

Gladiolus corms or stolons were collected (50 mg) and ground to a powder with liquid nitrogen. The extraction procedure was performed as described by Wu et al.^37^. d_6_-ABA and d_2_-GA_3_ were used as internal standards. The extracts were analyzed by HPLC-MS/MS^70^. Three biological replicates were examined.

### PAS staining

Gladiolus corms and stolons were fixed in formalin-aceto-alcohol (FAA) solution. The dehydration and embedding procedures were performed according to a previously published protocol^71^. Paraffin sections (8 μm) were obtained using a slicing machine. The slides were stained with PAS^72^ and photographed by a light microscope (Olympus BX51, Tokyo, Japan). Sugars were stained in red by PAS.

### Virus-induced gene silencing in Gladiolus

Silencing of the target gene (*GhSUS2*, *GhNCED*, or *GhGA20ox*) by VIGS was performed as described by Zhong et al.^73^ with some modifications. Briefly, a 200–500 base pair (bp) fragment specific to the target gene was generated and cloned into the pTRV2 vector (primers are listed in Table S1). The TRV1, TRV2, and TRV2 target genes were transformed individually into the Agrobacterium GV3101 strain. The transformed colonies were cultured overnight in LB medium containing 50 mg/L kanamycin and 50 mg/L rifampicin. Then, bacteria were collected and resuspended in infiltration buffer (10 mM MgCl_2_, 200 mM acetosyringone, and 10 mM 2-(N-morpholino) ethanesulfonic acid (pH 5.6)) to a final OD_600_ of 1.8. Equal volumes of TRV1 and TRV2 (the control), as well as the TRV1 and TRV2 target genes, were mixed together and kept in the dark for 3 h at 25 °C before vacuum infiltration. Corms were submerged in infiltration buffer and infiltrated under 0.9 MPa for 30 min. Ultimately, the corms were planted in pots and grown in a green chamber at 22 °C under 16/8 h light/dark for four months.

### Iodine staining of starch

Gladiolus leaves were submerged in 75% (v/v) ethanol at 80 °C until chlorophyll was eliminated. Then, the leaves were washed with ddH_2_O and stained with Lugol’s solution [0.06% I_2_ (w/v), 0.1% KI (w/v), and 4 mM HCl] for 10 min. Finally, the stained leaves were rinsed with ddH_2_O for 15 min^33^.

### RNA extraction and qRT-PCR

Total RNA from Gladiolus samples was extracted using the Tiangen RNA Extraction Reagent Kit (Tiangen, Beijing, China) and reverse transcribed with the M-MLV Reverse Transcriptase Kit (TaKaRa, Shiga, Japan). Approximately 400 ng of cDNA was used as the template for qRT-PCR and analyzed by using the Applied Biosystems StepOnePlus^TM^ real-time PCR system with the Takara qRT-PCR kit. The Gladiolus actin gene acted as the reference gene^45^. The PCR procedures used were based on the manufacturer’s instructions. All primers are listed in Table S1, and the data were analyzed with the 2^-ΔΔT^ method^74^.

### Subcellular localization of GhSUS2-GFP

The coding sequence of *GhSUS2* was cloned into pCAMBIA1300-GFP with the S*al*I and *Kpn*I restriction sites (pSuper: GFP-GhSUS2). Both the fusion construct (GFP-GhSUS2) and the control (empty vector; GFP) were transiently transformed into onion epidermal cells by particle bombardment. After incubation at 25 °C in the dark overnight, the cells were visualized using confocal microscopy (Zeiss LSM 710, Baden-Württemberg, Germany; 488 nm excitation and a 515–535 nm bandpass filter).

### Activities of SUS, INV, and SSS

Gladiolus corms were extracted by grinding tissue in liquid nitrogen before adding 1 ml of extraction buffer [25 mM HEPES-KOH (pH 7.3), 5 mM ethylenediamine tetraacetic acid (EDTA), 0.1% (w/v) polyvinyl pyrrolidone (Mr 4000), 1 mM dithiothreitol, 0.01 mM leupeptin and 1 mM phenylmethylsulfonyl fluoride]. The extraction procedures were performed as described by Fan et al*.*^69^. The supernatants were used for the determination of cytosolic invertase activity as described by Wang et al.^75^. SUS activity assays, in the direction of sucrose breakdown, were carried out as described by Wang et al*.*^76^. Starch synthase activity was measured using the Soluble Starch Synthase Activity Assay Kit (Solarbio, Beijing, China).

## Supplementary information

Supplementary tables and figures
