# Supplementary material for: Antagonism between abscisic acid and gibberellin regulates starch synthesis and corm development in Gladiolus hybridus
Source: Hortic Res. 2021 Jul 1;8:155. doi: 10.1038/s41438-021-00589-w (PMC8245626; doi:10.1038/s41438-021-00589-w)
Supplement: Supplementary file 1 — Supplementary tables and figures [file 41438_2021_589_MOESM1_ESM.docx]

| **Table S1 Primer sequences used in this study** | | |
| --- | --- | --- |
| **Primer** | **Forward sequence (5’-3’)** | **Reverse sequence (5’-3’)** |
| **CDS cloning** |  |  |
| GhSUS2-CDS | ATGGGCTCACTGAATCTCGCTCATAG | TTATTTCGCACCATTCGCAGCAG |
| **Virus-induced gene silencing (VIGS)** | | |
| TRV1 | TTACAGGTTATTTGGGCTAG | CCGGGTTCAATTCCTTATC |
| TRV2 | TGGGAGATGATACGCTGTT | CCTAAAACTTCAGACACG |
| GhNCED/TRV2 | GCTCTAGAGTGGTCGTGATCGGATCCTC | GGGGTACCCTCGCCTCCGTACTTGTTGT |
| GhGA20ox/TRV2 | CGGAATTCAATAAACCAACGTGGTCTTATTCCAC | GGGGTACCTTCGAGCTTGTGGGGCCGCT |
| GhSUS2/TRV2 | GCTCTAGAAGACTCGACGTTATCTCGAGATG | CGAGCTCGAAACTGCAAAGTTTACCAGAC |
| **qRT-PCR** |  |  |
| GhACTIN-QRT | ACTGCAGAGCGGGAAATTGT | CCAATCAGGGATGGCTGGAA |
| GhSUS2-QRT | CATAGCGCTCGGGATAGCTT | TCAGCTATGAGATGATGGGGC |
| GhCIN-QRT | ACCGACACCCGAATTTCCC | ACGTAAACGTCGAAGGGGTT |
| GhAPS-QRT | TAATCTCTCCTCCGGATCCTCC | CCAGACACGTCTGCGAGTT |
| GhNCED-QRT | CTCCGACCCTTCCGTTCAAA | TCACCGTCGAAGAAGTGGTG |
| GhGA20ox-QRT | GCCCACACGGATCGATTCTC | TGCCATTTGCCATCGACGAA |
| **Subcellular localization/ GhSUS2- overexpressed lines** | | |
| GhSUS2/pCAMBIA1300-GFP | ACGCGTCGACATGGGCTCACTGAATCTCGCTCATAG | CGGGGTACCTTATTTCGCACCATTCGCAGCAG |
|  |  |  |
|  |  |  |
|  |  |  |
|  |  |  |

**Table S2 Accession numbers used in this study**

| **symbol** | **species** | **accession number** |
| --- | --- | --- |
| CcSUS4-2 | *Theobroma cacao* | XM_007012485.1 |
| CcSUS4-1 | *Theobroma cacao* | XM_007012484.1 |
| GaSUS1 | *Gossypium arboreum* | HQ680462.1 |
| GaSUS2 | *Gossypium arboreum* | HQ680463.1 |
| GaSUS4 | *Gossypium arboreum* | HQ680465.1 |
| GaSUS5 | *Gossypium arboreum* | HQ680466.1 |
| GaSUS3 | *Gossypium arboreum* | HQ680464.1 |
| GhSUS1 | *Gossypium hirsutum* | FJ713478.1 |
| DlSUS1 | *Dimocarpus longan* | KP769776.1 |
| CiSUS1 | *Citrus unshiu* | AB022092.1 |
| BlSUS2 | *Betula luminifera* | AGV22112.1 |
| PtoSUS2 | *Populus tomentosa* | GU559728.1 |
| PtrSUS2 | *Populus tremuloides* | EU195082.1 |
| PtrSUS2 | *Populus trichocarpa* | GU559730.1 |
| HbSUS3 | *Hevea brasiliensis* | KC492045.1 |
| HbSUS4 | *Hevea brasiliensis* | KC492046.1 |
| HbSUS1 | *Hevea brasiliensis* | KC832927.1 |
| AtSUS4 | *Arabidopsis thaliana* | AT3G43190 |
| AtSUS1 | *Arabidopsis thaliana* | AT5G20830 |
| BvSUS2 | *Beta vulgaris* | AY457173.1 |
| DcSUS1 | *Dianthus caryophyllus* | AB543810.1 |
| GhSUS2 | *Gladiolus hybridus* | KP698512.1 |
| OhSUS1 | *Oncidium hybrid* | AAM95943.1 |
| BoSUS3 | *Bambusa oldhamii* | AF412037.1 |
| BoSUS1 | *Bambusa oldhamii* | AF412036.3 |
| ZmSH1 | *Zea mays* | NM_001111941.1 |
| ZmSUS1 | *Zea mays* | NM_001279762 |
| OsSUS2 | *Oryza sativa* | JF969237.1 |
| BoSUS2 | *Bambusa oldhamii* | AAL50571.1 |
| AtSUS2 | *Arabidopsis thaliana* | AT5G49190 |
| PpSUS2 | *Prunus persica* | KJ493331.1 |
| AhSUS2 | *Amaranthus hypochondriacus* | JQ012919.1 |
| PpSUS1 | *Pyrus pyrifolia* | BAB20799.1 |
| AtSUS6 | *Arabidopsis thaliana* | AT1G73370 |
| AtSUS5 | *Arabidopsis thaliana* | AT5G37180 |

**
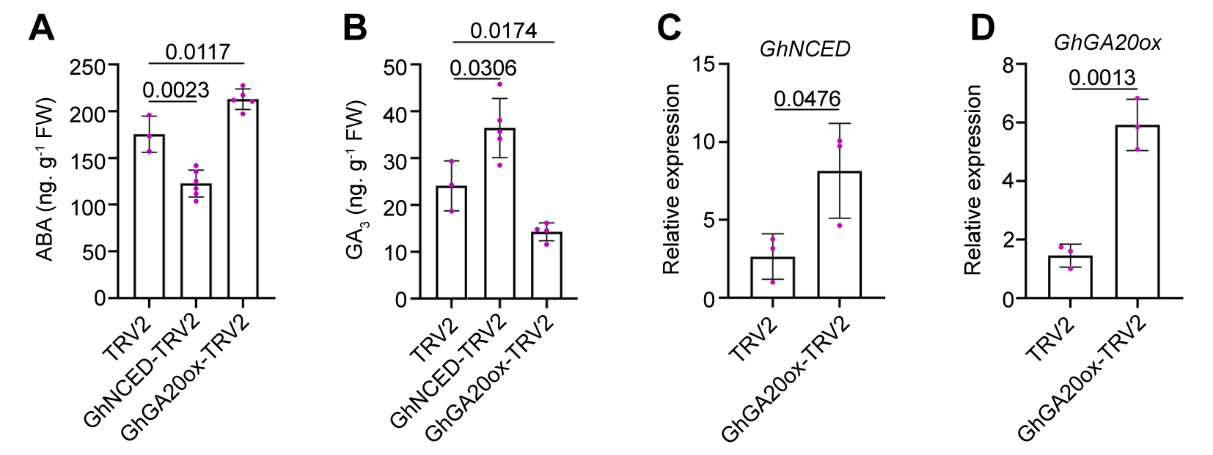
**

**Supplementary Figure 1 The endogenous ABA and GA_3_ in silenced corms. A**. ABA was decreased in *GhNCED*-silenced corms and was increased in *GhGA20ox*-silenced corms. **B**. GA_3_ was decreased in *GhGA20ox*-silenced corms and was increased in *GhNCED*-silenced corms. **C**. The transcript level of *GhNCED* was increased in *GhGA20ox*-silenced corms. **D**. The transcript level of *GhGA20ox* was increased in *GhNCED*-silenced corms. Averages of at least three biological replicates ± SD are shown. The significant differences were analyzed with the two-side paired t-test. P value was indicated above the black line.

**
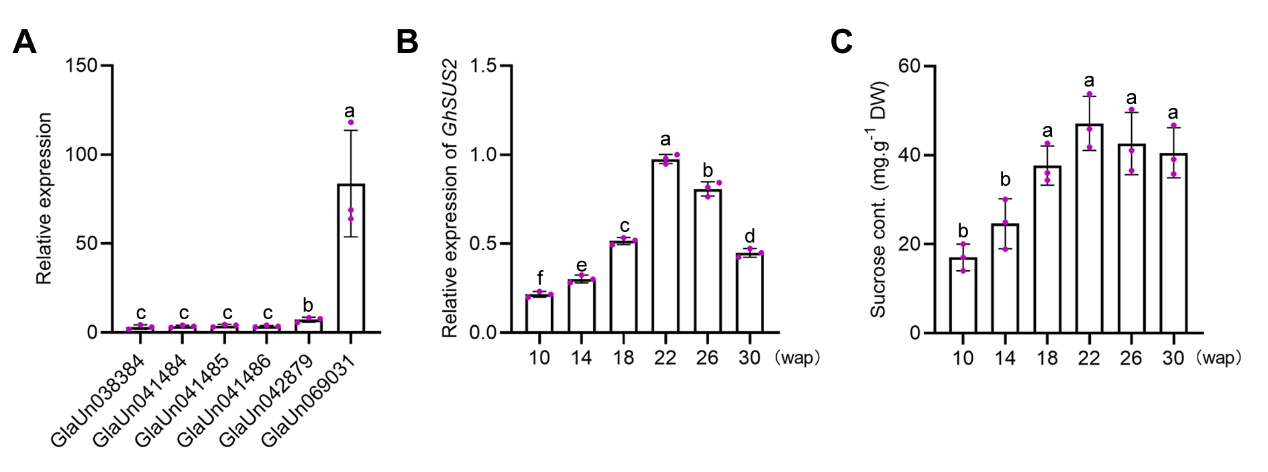
**

**Supplementary Figure 2 The expression pattern of *GhSUS2* and other members in Gladiolus. A**. The expression profile of *GhSUSs* in corms. *GhSUS2* (GlaUn069031) showed the highest transcript level among them. **B**. The expression pattern of *GhSUS2* in the cormels during the growth and harvesting stages. Cormels were formed at 10 weeks after planting (WAP) and were harvested at 27 WAP. **C**. sucrose was acuminate when corms were expanded (10-26 WAP). Averages of three biological replicates ± SD (n = 3) are shown. Different letters represent statistically significant differences at p < 0.05 (one-way ANOVA and Tukey HSD post-hoc test).


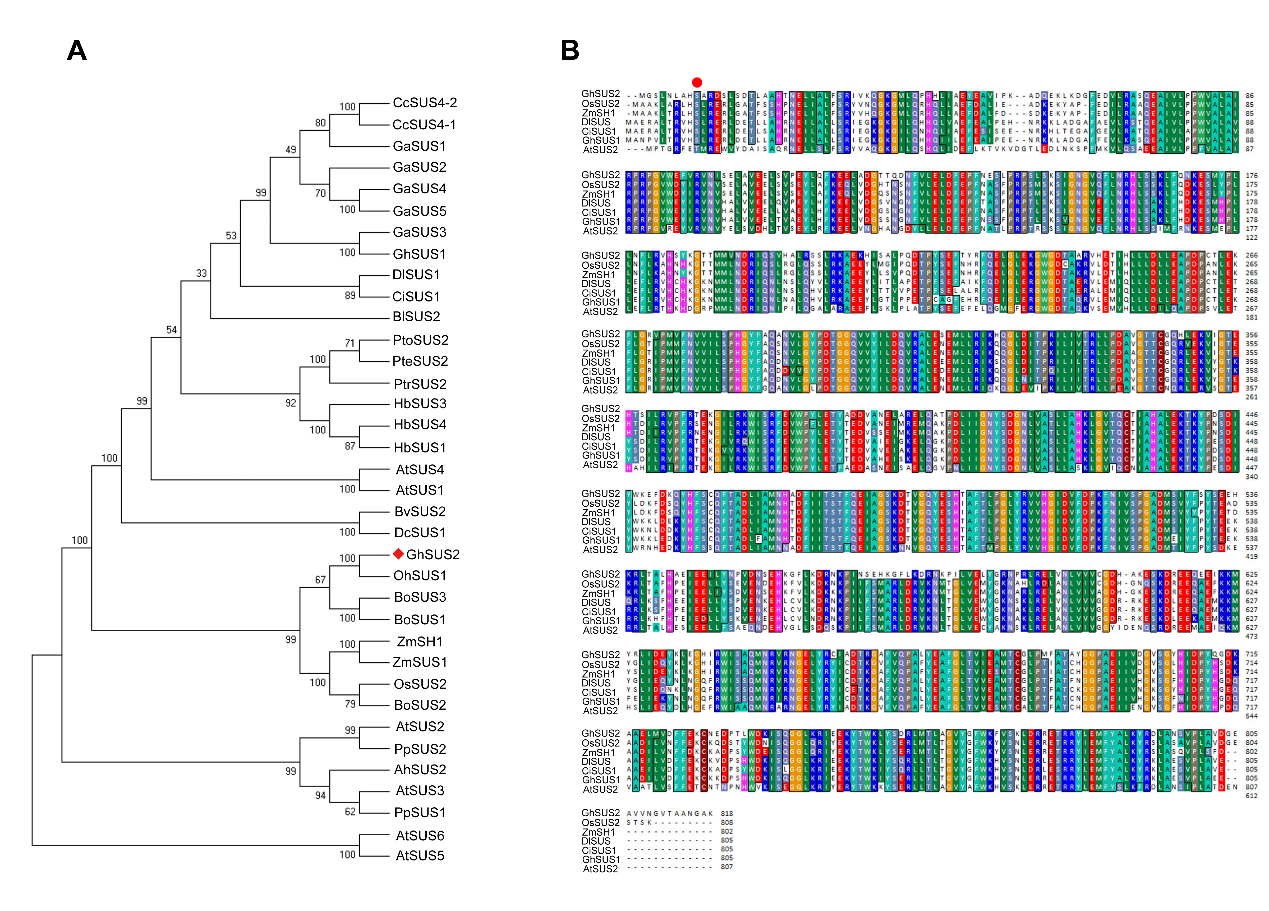


**Supplementary Figure 3 phylogenetic relationship and Sequence alignment of GhSUS2 with other homologous. A**. The phylogenetic tree of GhSUS2 protein and other homologous proteins from dicots and monocots. **B**. The sequence alignment of GhSUS2 with other homologous proteins. The red dot represents the conserved Ser-phosphorylation site in the N terminal. The accession number of homologous proteins were listed in Table S2.


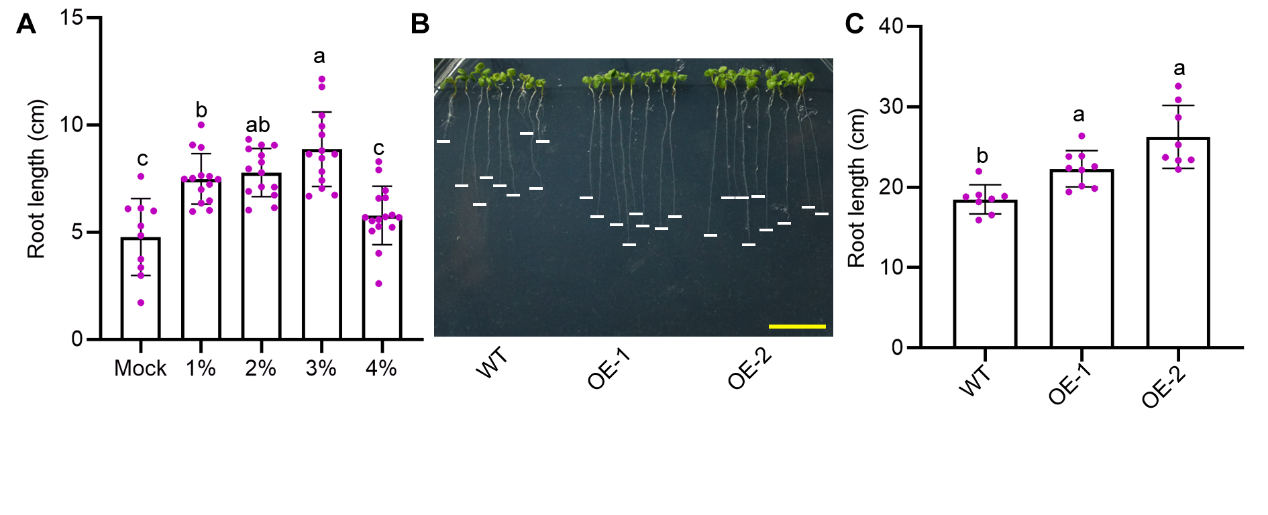


**Supplementary Figure 4 *GhSUS2* promotes the root elongation on sucrose media. A.** Root sensitivity to sucrose in *Col-0*. Roots had the best growth condition on MS media containing 3% (w/v) sucrose. Seeds of *Col-0* were spotted on MS media containing different concentrations of sucrose. After 3 days of chilling treatment, the plates were placed in the growth chamber for another 3 days. The root length was calculated by Image J. **B**. Ectopic expression of GhSUS2 led to a longer root on MS media containing 3% sucrose. **C**. The root length of Col-0 and GhSUS2 overexpressed (henceforth OE) lines. Seeds of OE and *Col-0* (WT) were spotted on MS media containing 3% sucrose. After 3 days of chilling treatment, the plates were placed in the growth chamber for another 3 days. The root length was calculated by Image J. Bar = 1 cm. Three biological replicates were performed. The error bar represents SD. Different letters represent statistically significant differences at p < 0.05 (one-way ANOVA and Tukey HSD post-hoc test).

**Supplementary methods**

**phylogenetic relationship and Sequence alignment of GhSUS2**

The ExPASy Proteomics Tools ClustalX1.8^1^ and BioEdit7.0^2^ were used to perform multiple amino acid alignments, and a phylogenetic tree was constructed by the neighbor-joining method using the MEGA5.0 software^3^. Accession numbers for public sequences used in the phylogenetic analysis are listed in Supplemental Table S2.

**GhSUS2-overexpressed transgenic Arabidopsis**

The pSuper: GFP-GhSUS2/ pCAMBIA1300 construct was transformed into the GV3101 agrobacteria strain. Arabidopsis of *Col-0* background was transformed using the floral dip method^4^. The harvested seeds were sterilized and selected on MS media containing 50 mg/L hygromycin (Roche) before 3-day stratification. The plates were plated in the growth chamber at 22**°C** and 16/8 h light/ dark. The resistant plants were genotyped by PCR with primers listed in Table S1. Homozygous plants were used in the assay.

**Supplementary references**

1. Thompson, J.D., Gibson, T.J., Plewniak, F., Jeanmougin, F. & Higgins, D.G. The CLUSTAL_X windows interface: flexible strategies for multiple sequence alignment aided by quality analysis tools. *Nucleic Acids Res* **25**, 4876-82 <http://dx.doi.org/10.1093/nar/25.24.4876> (1997).

2. Hall, T.A. BIOEDIT: A user-friendly biological sequence alignment editor and analysis program for windows 95/98/ NT. (1999).

3. Tamura, K. *et al.* MEGA5: molecular evolutionary genetics analysis using maximum likelihood, evolutionary distance, and maximum parsimony methods. *Mol Biol Evol* **28**, 2731-2739 <http://dx.doi.org/10.1093/molbev/msr121> (2011).

4. Clough, S.J. & Bent, A.F. Floral dip: a simplified method for Agrobacterium-mediated transformation of Arabidopsis thaliana. *Plant J* **16**, 735-43 <http://dx.doi.org/10.1046/j.1365-313x.1998.00343.x> (1998).
